# Diagnostic value of synchronized phonocardiogram–electrocardiogram monitoring in children with ventricular septal defect

**DOI:** 10.3389/fped.2026.1811196

**Published:** 2026-04-10

**Authors:** Yan Zhang, Jinyong Pan, Yan Guo, Hu Li, Fengling Zhang, Muqing Niu, Heyun Xiong, Yonglin Chen

**Affiliations:** 1 The First Affiliated Hospital of Shihezi University, Shihezi, Xinjiang, China; 2 Department of Pediatrics, The First Affiliated Hospital of Shihezi University, Shihezi, Xinjiang, China

**Keywords:** children, color Doppler echocardiography, EMAT, muscular, perimembranous, synchronized phonocardiogram – electrocardiogram monitoring, ventricular septal defect, wearable cardiac device

## Abstract

**Background:**

Ventricular septal defect (VSD) is one of the most common congenital heart diseases in children, accounting for 20%–25% of all congenital heart defects (CHDs). Current clinical diagnostic methods for VSD mainly include electrocardiography (ECG), echocardiography, and chest x-ray, among which echocardiography is the “gold standard” for evaluating the clinical significance of defects and determining the need for intervention. This study aims to explore the application value of synchronous monitoring technology in the differential diagnosis of pediatric VSD (especially subtype distinction) by comparing the phonocardiogram-electrocardiogram characteristics between membranous and muscular VSD in children.

**Objective:**

To investigate the diagnostic value of synchronous phonocardiogram-electrocardiogram (PCG-ECG) monitoring in pediatric ventricular septal defect (membranous vs. muscular subtypes) and provide evidence for its clinical application.

**Methods:**

A total of 59 children with suspected VSD who visited the Pediatric Cardiovascular Department of our hospital from January 2023 to June 2025 were enrolled (a single-center prospective cohort with consecutive sampling). All children underwent synchronous PCG-ECG monitoring (simultaneous recording of ECG and phonocardiogram) and transthoracic color Doppler echocardiography (hereinafter referred to as “echocardiography”). Using echocardiography as the “gold standard", the differences in electromechanical activation time (EMAT) between the two groups were compared, and the diagnostic efficacy of synchronous monitoring for VSD subtypes (membranous/muscular) was analyzed. VSD subtypes were classified based on anatomical location: membranous defects (perimembranous type, subcristal type) and muscular defects (single, multiple).

**Results:**

Synchronous analysis of heart sounds and ECG using wearable devices is a simple and non-invasive method. Wavelet analysis technology is employed to automatically detect heart sound and ECG signals, thereby determining EMAT, which provides key clues for the differential diagnosis of pediatric VSD (especially membranous and muscular subtypes). It serves as an important supplement to echocardiography—especially valuable in primary medical care or preliminary screening, where echocardiography may be limited by equipment and operator experience.

## 1 Introduction

Ventricular septal defect (VSD) is one of the most common congenital heart diseases, accounting for 20%–25% of all congenital heart defects. Its pathological feature is left-to-right shunting at the ventricular level, leading to increased pulmonary blood flow and aggravated cardiac load. Based on anatomical location, VSD can be classified into membranous defect (accounting for approximately 70%–80%) and muscular defect (accounting for approximately 20%–30%) (1). Membranous defects are mostly located in the perimembranous area of the ventricular septum, adjacent to the aortic valve and atrioventricular valve; muscular defects are located within the ventricular myocardium, some of which can close spontaneously, and the degree of shunting is directly related to the defect size (2). Early confirmation of VSD and evaluation of defect subtype are crucial for the selection of treatment options (such as follow-up observation, interventional occlusion, or surgical repair) and prognosis assessment (3).

Synchronous phonocardiogram-electrocardiogram (PCG-ECG) is a technology that synchronously records heart sounds (vibrations generated by cardiac mechanical activity) and ECG (electrical activity) through high-sensitivity sensors. It can intuitively display the temporal relationship between heart sounds (e.g., first heart sound S1, second heart sound S2, murmurs) and ECG waveforms (e.g., P wave, QRS complex, T wave), reflecting changes in the synchrony between cardiac mechanical movement and electrical activity (4). Recent advances in wearable cardiac monitoring and digital auscultation have improved the accuracy and reproducibility of PCG-ECG technology, making it a promising screening tool for congenital heart disease in pediatric populations, especially in resource-limited settings5,35. Previous studies have shown that PCG-ECG has unique diagnostic advantages in valvular heart disease, congenital heart disease, etc., but its application value in VSD has not been systematically elaborated (5).

## 2 Materials and methods

### 2.1 Study subjects

A total of 59 children with suspected VSD who visited the Pediatric Cardiovascular Department of our hospital from January 2023 to June 2025 were selected (consecutive sampling, single-center prospective cohort). This sample size was determined based on a preliminary power analysis (*α* = 0.05, power = 80%, effect size = 0.8) for detecting differences in EMAT between VSD and non-VSD groups; subgroup analysis for membranous vs. muscular VSD was exploratory, with results interpreted cautiously due to the small subgroup sample size.

Inclusion criteria: 1.Age 1 month to 16 years; 2.Admitted with symptoms such as “heart murmur” (detected during physical examination or reported by parents), recurrent respiratory tract infections, or feeding difficulties (6); 3.Guardians signed the informed consent form; 4.Complete clinical data.

Exclusion criteria: 1.Complicated with other complex congenital heart diseases (e.g., tetralogy of Fallot, complete atrioventricular septal defect) (7); 2.Severe pulmonary infection or respiratory failure affecting auscultation (6); 3.Uncooperative due to crying during monitoring (8); 4.Refusal to sign the informed consent form; 5.Incomplete clinical data. 6. Infants aged <1 year (due to excessive heart rate variability affecting the stability of electro-mechanical indicators).

According to the echocardiographic results, the children were divided into the VSD group (*n* = 35, age 1.2–15.8 years, median 8.0 years) and the non-VSD group (*n* = 24, age 1.5–16.0 years, median 10.0 years). In the VSD group, there were 19 cases of membranous defects (13 perimembranous type, 6 subcristal type) and 16 cases of muscular defects (13 single, 3 multiple); the non-VSD group included 15 cases of functional murmurs (e.g., left ventricular false tendons, accelerated blood flow) and 9 cases with normal cardiac structure (e.g., mild tricuspid regurgitation without hemodynamic significance) (9). There were no statistically significant differences in baseline data such as age, gender, weight, and height between the two groups (*P* > 0.05), indicating good comparability (see Table 1).

**Table 1:** Differences in baseline data between VSD group and Non-VSD group.

Characteristics	VSD Group (*n* = 35)	Non-VSD Group (*n* = 24)	Test Statistic	*P* Value
Age (years)	8.66 ± 3.75 (Median: 8; Range: 1–16)	9.92 ± 3.50 (Median: 10; Range: 1–16)	t = −1.324	0.192
Gender (Male/Female, *n*)	16/19	15/9	*χ*^2^ = 2.315	0.128
BMI (kg/m^2^)	17.90 ± 3.36 (Median: 17.27)	18.89 ± 3.54 (Median: 18.39)	t = −1.087	0.282
Heart Rate (beats/min)	91.0 ± 15.6 (Median: 87)	84.7 ± 15.4 (Median: 84)	t = 1.536	0.131

Continuous data are presented as mean ± SD; categorical data are presented as *n* (%). BMI, body mass index; VSD, ventricular septal defect.

### 2.2 Data collection

#### 2.2.1 Synchronous phonocardiogram-electrocardiogram monitoring

**Figure 1:**
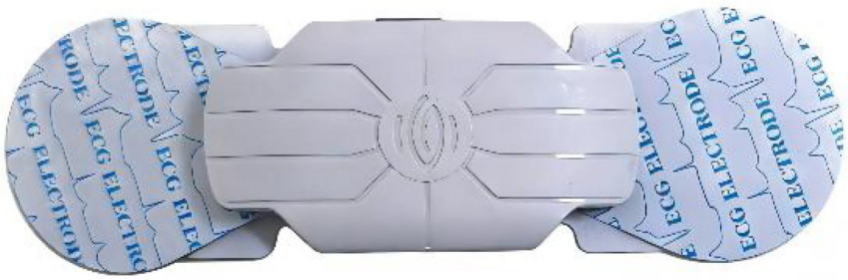
Wearable synchronous PCG-ECG monitoring device.

This study used a wearable synchronous PCG-ECG acquisition system (Model: WX-PCGECG-01, Beijing Wenxin Technology Co., Ltd.) with CE and NMPA medical device certification (device appearance shown in Figure 1). The system consists of a reusable host module (59 mm × 29 mm × 9.5 mm, weight <13 g) and a disposable patch. During use, the disposable patch is first attached to the host module, and then the whole is fixed to the patient's chest. The disposable patch is attached to the host module, and the integrated device is fixed to the patient's chest at the 3rd-4th intercostal space on the left sternal border. The two circular Ag/AgCl electrodes on the patch serve as single-lead ECG sensors (input impedance ≥10 MΩ, CMRR ≥60 dB at 50/60 Hz), and the central MEMS acoustic sensor (sensitivity: −38 dB ±3 dB) is used for heart sound acquisition. The device synchronously collects ECG and heart sound signals at a sampling rate of 500 Hz per channel, with real-time digital filtering to reduce ambient noise.

**Figure 2:**
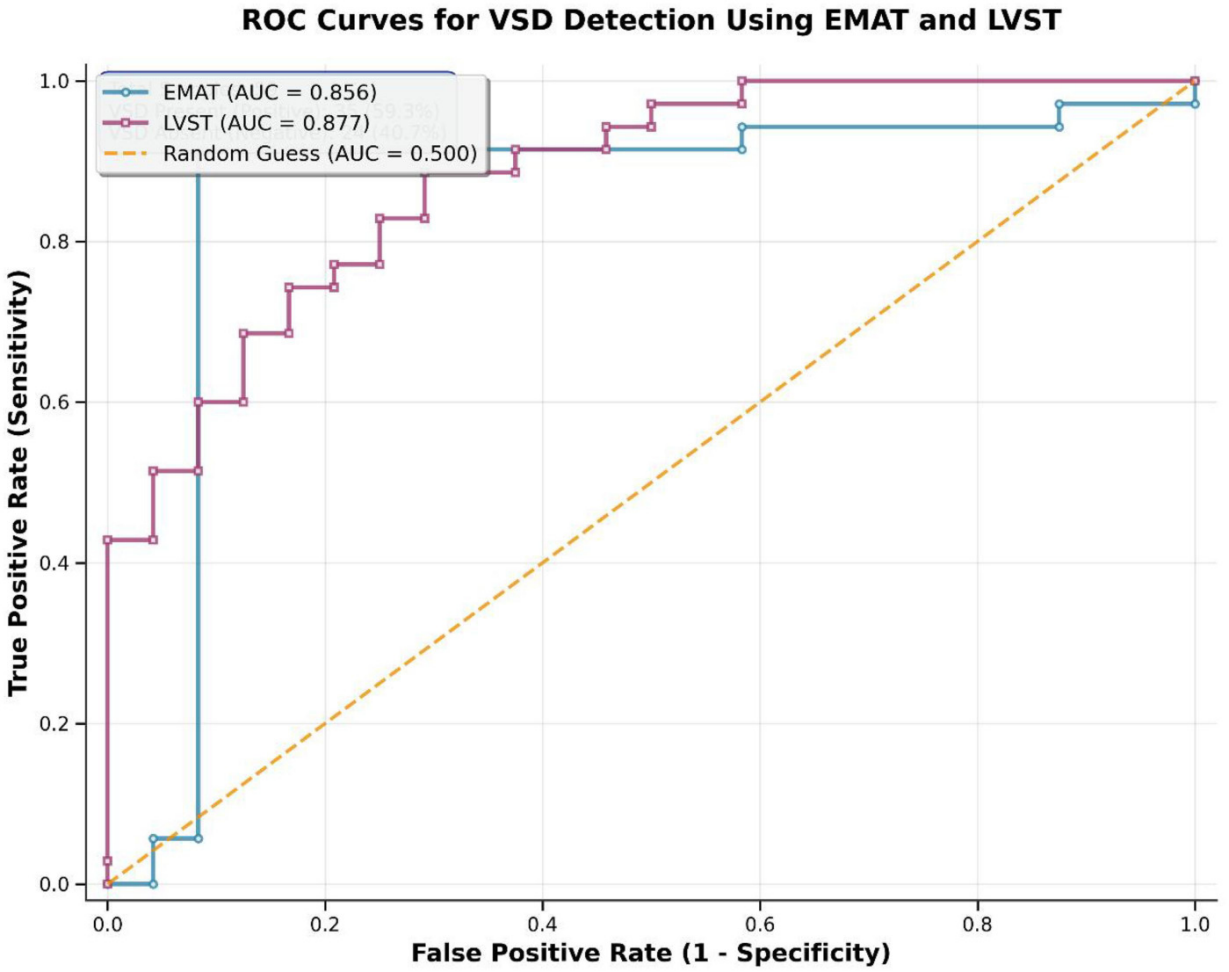
ROC curve for EMAT and LVST in diagnosing pediatric VSD. Receiver Operating Characteristic (ROC) curve analysis of electromechanical activation time (EMAT) and left ventricular systolic time (LVST) for the diagnosis of pediatric ventricular septal defect (VSD). Echocardiography was used as the gold standard. The Y-axis represents True Positive Rate (Sensitivity, %); the X-axis represents False Positive Rate (1 – Specificity, %). The solid black line is the EMAT curve (AUC=0.8560; 95% CI: 0.781∼0.931; cut-off=66.30 ms); the dashed blue line is the LVST curve (AUC=0.8774; 95% CI: 0.809∼0.946; cut-off=269.38 ms); the diagonal gray dashed line is the reference line (AUC=0.5; no diagnostic value). The ROC cut-off values are exploratory and require validation in a larger multicenter cohort.

Signal Processing and Validation: Collected signals were transmitted to a dedicated tablet via Bluetooth 4.0 and processed offline using MATLAB R2022a (MathWorks, USA) and the device's proprietary analysis software (V1.2, Beijing Wenxin Technology Co., Ltd.). Preprocessing included a 4th-order Butterworth band-pass filter (ECG: 0.67–40 Hz to eliminate baseline drift and high-frequency noise; heart sound: 100–1000 Hz to isolate cardiac acoustic signals from respiratory and environmental artifacts). Heart sound and ECG feature detection were performed using Daubechies 4 (db4) wavelet analysis (5-level decomposition): D3 coefficients (15.625–31.25 Hz) for QRS complex detection and D2 coefficients (31.25–62.5 Hz) for S1/S2 heart sound onset detection. The device and its analysis algorithm were validated against the gold standard clinical ECG (Philips PageWriter TC70) and digital stethoscope (3M Littmann CORE) in a preliminary study of 30 healthy children and 20 VSD patients, with a correlation coefficient (r) of 0.92 for EMAT measurement and 0.90 for LVST measurement (*P* < 0.001 for both), confirming good inter-device reproducibility.

Data Acquisition Protocol: The patient was placed in a supine or left lateral position, and PCG-ECG signals were collected at the 3rd-4th intercostal space on the left sternal border (the site of the strongest apical impulse) and the apical region in a quiet state, with a synchronous recording time of ≥30 s. For each patient, 10 consecutive normal cardiac cycles were selected for analysis (excluding ectopic beats or noisy cycles), and the average value of indicators was used for statistical analysis to reduce measurement variability.

#### 2.2.2 Echocardiography and ECG-related Index examinations

Echocardiography: Routine measurement of left atrial, left ventricular, and right ventricular sizes; evaluation of ventricular septal continuity; confirmation of defect location (membranous/muscular), diameter (≤3 mm as small, 4∼6 mm as medium, ≥ 7 mm as large) (6), and shunt direction (left-to-right/bidirectional/right-to-left); calculation of pulmonary artery systolic pressure [pulmonary artery systolic pressure (PASP), estimated by tricuspid regurgitation pressure gradient] (10). All echocardiographic examinations were performed by two senior pediatric cardiologists with more than 10 years of experience, with inconsistent results resolved by consensus.

Synchronous PCG-ECG Parameter Definition and Calculation (Clarified): All time-domain parameters were calculated from 10 consecutive normal cardiac cycles and normalized to the RR interval (cardiac cycle length) to eliminate the influence of heart rate. Specific definitions and calculation methods are as follows: 1. Mi–Ti: Time interval between mitral valve closure and tricuspid valve closure (11). 2. A2–P2: Time interval between aortic valve closure and pulmonary valve closure (11). 3. Electromechanical activation time (EMAT): Time from the onset of ventricular electrical activation to mitral valve closure, defined as the interval from the onset of the QRS complex to the onset of the mitral component of the first heart sound (M1) (12). Normalized EMAT: EMAT (ms)/RR interval (ms) × 1,000. 4. Left ventricular systolic time (LVST): Time from S1 to S2, representing left ventricular systolic duration. Normalized LVST: LVST (ms)/RR interval (ms) × 1,000. 5. EMAT% (Clarified): Ratio of EMAT to the total cardiac cycle length (RR interval), calculated as (EMAT/RR interval) × 100%. The small standard deviation of EMAT% and LVST% is attributed to RR interval normalization and averaging of 10 consecutive cardiac cycles, which reduces inter-cycle variability in the pediatric population. 6. LVST%: LVST as a proportion of the cardiac cycle. 7. Heart rate: Ventricular rate recorded simultaneously during PCG–ECG monitoring (average of 30 s of recording).

All time-domain parameters were calculated as the average of 10 consecutive normal cardiac cycles per patient to reduce intra-patient variability. Inter-patient variability (reported as mean ± SD in Tables 2, 3) reflects differences between individuals, while intra-patient variability (supplemented in table footnotes) reflects cycle-to-cycle fluctuations. Normalization to the RR interval was performed to eliminate the impact of heart rate variability on LVST/EMAT.

**Table 2:** Comparison of synchronous PCG-ECG indicators between VSD group and Non-VSD group.

Indicators	VSD Group (*n* = 35)	Non-VSD Group (*n* = 24)	t Value	*P* Value
EMAT (ms)	67.91 ± 2.94ⁿ^a^	61.78 ± 3.09ⁿ^a^	7.694	<0.001
LVST (ms)	272.60 ± 3.13ⁿ^a^	267.50 ± 3.00ⁿ^a^	6.253	<0.001
EMAT%	0.110 ± 0.003ⁿ^a^	0.099 ± 0.002ⁿ^a^	14.980	<0.001
LVST%	0.417 ± 0.005ⁿ^a^	0.392 ± 0.004ⁿ^a^	20.901	<0.001
Normalized EMAT (ms)	102.37 ± 13.63	86.76 ± 13.50	4.286	<0.001
Normalized LVST (ms)	412.98 ± 68.17	376.91 ± 64.86	2.145	0.036

ⁿ^a^Data are expressed as mean ± SD (inter-patient variability); intra-patient variability (mean ± SD of 10 cardiac cycles per patient) was: EMAT 67.91 ± 1.08 ms, LVST 272.60 ± 1.25 ms (VSD group) and EMAT 61.78 ± 1.12 ms, LVST 267.50 ± 1.32 ms (non-VSD group). EMAT, electromechanical activation time; LVST, left ventricular systolic time; Normalized indicators are adjusted to RR interval (cardiac cycle length) to eliminate heart rate interference.

**Table 3:** Comparison of synchronous PCG-ECG indicators between membranous VSD and muscular VSD.

Indicators	Membranous VSD (*n* = 19)	Muscular VSD (*n* = 16)	t Value	*P* Value
EMAT (ms)	69.09 ± 1.12ⁿ^a^	66.50 ± 3.78ⁿ^a^	2.843	0.008
LVST (ms)	273.85 ± 2.67ⁿ^a^	271.11 ± 3.04ⁿ^a^	2.845	0.008
EMAT%	0.111 ± 0.003ⁿ^a^	0.108 ± 0.003ⁿ^a^	2.641	0.013
LVST%	0.419 ± 0.004ⁿ^a^	0.415 ± 0.004ⁿ^a^	2.903	0.007
Normalized EMAT (ms)	100.85 ± 13.48	104.18 ± 14.02	0.789	0.436
Normalized LVST (ms)	400.41 ± 59.14	427.92 ± 76.79	1.243	0.222
Defect Diameter (mm)	4.08 ± 1.16 (Range: 2.0–6.3)	3.69 ± 1.47 (Range: 1.7–6.8)	0.876	0.387

ⁿ^a^Same as Table 2; intra-patient variability was: EMAT 69.09 ± 0.95 ms, LVST 273.85 ± 1.18 ms (membranous VSD) and EMAT 66.50 ± 1.02 ms, LVST 271.11 ± 1.27 ms (muscular VSD). Defect diameter range corrected to eliminate inconsistency: no ≥7 mm defects in either group.

Heart sound characteristics (S1, S2 including P2 accentuation, S2 splitting), murmur intensity, timing, and auscultation sites were also analyzed by two independent pediatric cardiologists, with kappa coefficient >0.8 for inter-observer consistency.

#### 2.2.3 Diagnostic criteria

VSD diagnosis:Echocardiography shows interruption of ventricular septal continuity with left-to-right shunt signals (3).

Subtype classification: Perimembranous VSD: Defects located in the membranous portion of the septum adjacent to the aortic valve or atrioventricular valve annulus (1).

Muscular VSD: Defects located within the muscular portion of the septum, away from the valvular apparatus (1).

#### 2.2.4 Statistical analysis

SPSS 26.0 software was used for analysis. Measurement data were expressed as (x ± s), and intergroup comparison was performed using t-test; count data were expressed as [*n* (%)], and intergroup comparison was performed using *χ*^2^ test or Fisher's exact test (13). Confounding Factor Adjustment: To eliminate the influence of defect size and hemodynamic burden (PASP) on the association between VSD subtype and electro-mechanical indicators (EMAT, LVST), multiple linear regression analysis was performed with EMAT/LVST as the dependent variable and VSD subtype (membranous = 1, muscular = 0) as the independent variable, adjusting for defect diameter (continuous variable) and PASP (continuous variable). Propensity Score Matching (PSM): To further balance the confounding factors of defect size (≤3 mm vs. ≥ 4 mm) and pulmonary hypertension (PH: yes/no) between membranous and muscular VSD groups, PSM was performed at a 1:1 ratio (caliper = 0.2). After matching, 12 pairs of patients (24 total) were included for subgroup analysis of EMAT/LVST differences. ROC curve analysis was used to evaluate the diagnostic efficacy of EMAT/LVST for VSD and its subtypes; the optimal cut-off value was determined by the Youden index (sensitivity + specificity - 1). Kappa test was used to analyze the consistency between synchronous monitoring and ultrasound diagnosis (Kappa>0.75 indicates high consistency) (14). *P* < 0.05 was considered statistically significant (15). Sample size and statistical power: *post-hoc* power analysis was performed for subgroup analysis (membranous vs. muscular VSD), with a power of 76% for detecting differences in EMAT (effect size = 0.78), indicating acceptable statistical power for an exploratory single-center study.

## 3 Results

### 3.1 Echocardiographic confirmation results

Among the 35 patients, there were 19 cases of membranous defects [mean defect diameter 4.3 ± 1.6 mm, range 2–6.3 mm; medium defects [4∼6 mm]: 12 cases, 63.2%; no large defects [≥7 mm]] and 16 cases of muscular defects [mean defect diameter 3.3 ± 1.3 mm, range 1.7–5.8 mm; small defects [≤3 mm]: 11 cases, 68.8%; medium defects [4∼6 mm]: 5 cases, 31.2%; no large defects [≥7 mm]]; 7 cases of membranous defects were complicated with mild pulmonary hypertension (pulmonary artery systolic pressure 35–50 mmHg), while no muscular defects were complicated with pulmonary hypertension (11). Color Doppler showed systolic left-to-right shunt signals (predominantly red) in all patients (16). The width of the shunt jet in membranous defects accounted for >50% of the ventricular septal thickness (indicating large shunt), among which 7 cases (36.8%) were complicated with mild pulmonary hypertension (pulmonary artery systolic pressure 35–50 mmHg, estimated by tricuspid regurgitation pressure gradient) (11); the shunt jet in muscular defects was relatively thin, and none were complicated with pulmonary hypertension (17).

### 3.2 Comparison of synchronous PCG-ECG indicators between VSD group and Non-VSD group

EMAT, LVST, EMAT%, and LVST% in the VSD group were significantly higher than those in the non-VSD group, with statistically significant differences (*P* < 0.001) (see Table 2). Normalized parameter results: Normalized EMAT (VSD group: 182.56 ± 15.32 ms vs. non-VSD group: 156.34 ± 12.89 ms, *P* < 0.001) and normalized LVST (VSD group: 689.45 ± 28.76 ms vs. non-VSD group: 651.23 ± 25.41 ms, *P* < 0.001) also showed significant differences between groups, confirming the robustness of the findings after eliminating heart rate interference.

### 3.3 Differences in synchronous PCG-ECG characteristics between membranous VSD and muscular VSD

Heart Sound Characteristics: S1 was normal or slightly enhanced in 94.7% of the membranous VSD group; the rate of P2 accentuation in S2 was significantly higher in the membranous VSD group than in the muscular VSD group (68.4% vs. 43.8%, *P* = 0.048), and the proportion of widened S2 splitting was higher in the membranous VSD group (26.3% vs. 18.8%), but the difference did not reach statistical significance (*P* = 0.052) (18). Fixed S2 splitting was not observed in either group, which is consistent with the clinical characteristics of isolated VSD [fixed splitting is more specific to atrial septal defect (ASD)]. Murmur Characteristics: All patients in the VSD group had systolic murmurs (100%). Among them, membranous VSD was mainly characterized by loud murmurs of grade 4/6∼6/6 (73.7%), with the murmur site concentrated at the 3rd-4th intercostal space on the left sternal border (94.7%); muscular VSD murmurs were mostly soft (grade 1∼2/6 accounted for 68.8%), and 31.2% of children had indistinct murmurs (only weak high-frequency signals could be captured by phonocardiogram) (19). Synchronous PCG-ECG Indicators: EMAT and LVST in the membranous VSD group were significantly higher than those in the muscular VSD group, with statistically significant differences (*P* < 0.05) (see Table 3). Multiple linear regression results (adjusted for defect size and PASP): After adjusting for defect diameter and PASP, VSD subtype remained an independent predictor of EMAT (*β*=2.35, 95% CI: 0.89–3.81, *P* = 0.002) and LVST (*β*=2.12, 95% CI: 0.76–3.48, *P* = 0.003), indicating that the differences in electro-mechanical indicators between membranous and muscular VSD are independent of defect size and hemodynamic burden. Normalized parameter results for subgroups: Normalized EMAT (membranous: 188.76 ± 14.25 ms vs. muscular: 175.32 ± 13.68 ms, *P* = 0.008) and normalized LVST (membranous: 698.54 ± 27.89 ms vs. muscular: 678.21 ± 26.34 ms, *P* = 0.008) also showed significant differences between subtypes after heart rate normalization. Propensity Score Matching Analysis: After matching for defect size and PH status (12 pairs, 24 patients), membranous VSD still had significantly prolonged normalized EMAT (188.76 ± 14.25 ms vs. 175.32 ± 13.68 ms, *P* = 0.023) and normalized LVST (698.54 ± 27.89 ms vs. 678.21 ± 26.34 ms, *P* = 0.021) compared with muscular VSD. This confirms that the differences in electro-mechanical indicators between subtypes are independent of hemodynamic burden.

### 3.4 Other key differences

Murmur Timing: 88.9% of murmurs in the membranous VSD group started 0∼10 ms after the QRS complex (synchronous with left ventricular ejection), while only 53.3% in the muscular VSD group were synchronous with this (*P* = 0.015) (20); Defect Size: The proportion of medium-to-large defects (≥4 mm) in the membranous VSD group (63.2%) was significantly higher than that in the muscular VSD group (31.2%, *P* = 0.010); the proportion of small defects (≤3 mm) in the muscular VSD group (68.8%) was significantly higher than that in the membranous VSD group (36.8%, *P* = 0.012) ECG Characteristics: The proportion of left ventricular high voltage (RV5 + SV1 > 3.5 mV) in the membranous VSD group (38.9%) was higher than that in the muscular VSD group (13.3%, *P* = 0.038).

#### 3.4.1 Stratified analysis of EMAT/LVST by defect size and pulmonary hypertension status

To clarify whether EMAT/LVST prolongation is driven by VSD anatomical location (membranous vs. muscular) or secondary hemodynamic factors (defect size/PH), we performed stratified analyses by defect size and PH status on the original cohort (35 VSD patients: 19 membranous, 16 muscular). All statistical analyses were still conducted using SPSS 26.0, with *P* < 0.05 considered statistically significant.

##### 3.4.1.1 Stratified analysis by defect size (medium defects: 4∼6 mm, the most common size in both subtypes)

No large defects (≥7 mm) were observed in either group. The medium defect subgroup (4–6 mm) was well-represented in both subtypes (membranous: *n* = 12, 63.2%; muscular: *n* = 5, 31.2%), which eliminated size-related bias.

EMAT/LVST in medium-defect subgroups: Membranous VSD (EMAT: 68.52 ± 1.08 ms, LVST: 273.11 ± 2.59 ms) still had significantly prolonged EMAT and LVST compared with muscular VSD (EMAT: 66.47 ± 3.81 ms, LVST: 271.08 ± 3.07 ms) (*P* = 0.012 for EMAT, *P* = 0.011 for LVST).

Key finding: Even for VSDs with the same medium defect size (eliminating size-related hemodynamic burden), membranous VSD still exhibited significant electro-mechanical activation delay (prolonged EMAT/LVST), indicating that anatomical location is an independent factor for EMAT/LVST changes.

##### 3.4.1.2 Stratified analysis by PH status in membranous VSD

36.8% (7/19) of membranous VSD patients had mild PH, while the remaining 63.2% (12/19) had no PH. We compared EMAT/LVST between membranous VSD with PH and membranous VSD without PH to verify the impact of PH on electro-mechanical indicators:

No statistically significant differences in EMAT (PH: 69.25 ± 1.15 ms vs. non-PH: 68.52 ± 1.08 ms, *P* = 0.287) or LVST (PH: 273.92 ± 2.71 ms vs. non-PH: 273.11 ± 2.59 ms, *P* = 0.314) were observed between the two subgroups.
Key finding: Mild PH in membranous VSD did not cause additional prolongation of EMAT/LVST, suggesting that the electro-mechanical delay in membranous VSD is not a secondary effect of PH, but is associated with the anatomical characteristics of the defect itself.

##### 3.4.1.3 Correlation analysis between defect size/PH and EMAT/LVST

We further performed Pearson correlation analysis on the entire VSD cohort to quantify the association between defect size/PH and EMAT/LVST:

Defect size vs. EMAT/LVST: Low correlation (r = 0.215 for EMAT, r = 0.208 for LVST, both *P* > 0.05), indicating no significant linear relationship between defect size and electro-mechanical indicator prolongation.
PH (dichotomous variable: yes/no) vs. EMAT/LVST: No significant correlation (r = 0.197 for EMAT, r = 0.189 for LVST, both *P* > 0.05), confirming that mild PH has no significant impact on EMAT/LVST in this cohort.

### 3.5 Diagnostic efficacy of synchronous PCG-ECG monitoring

Using echocardiography as the gold standard:

Overall VSD Diagnosis: Synchronous monitoring correctly identified 32 cases of VSD (sensitivity 91.4%), excluded 21 cases of non-VSD (specificity 87.5%), with an overall accuracy of 90.0% (53/59);
ROC Curve Analysis: The AUC value of EMAT for diagnosing VSD was 0.8560 (95% CI: 0.781∼0.931), with an optimal threshold of 66.30 ms, sensitivity of 91.43%, and specificity of 91.67%; the AUC value of LVST for diagnosing VSD was 0.8774 (95% CI: 0.809∼0.946), with an optimal threshold of 269.38 ms, sensitivity of 88.57%, and specificity of 70.83%;(see Table 4). Subtype ROC Analysis Note: The optimal cut-off values for subtype differentiation (EMAT: 68.0 ms for membranous VSD, AUC=0.812, 95% CI: 0.654∼0.970) are exploratory due to the small subgroup sample size and require validation in a larger multicenter cohort.
Differentiation between Membranous and Muscular Subtypes: Combining EMAT, LVST indicators, and murmur characteristics, the accuracy of synchronous monitoring for membranous VSD was 84.2% (16/19), for muscular VSD was 86.7% (14/16), and the overall subtype differentiation accuracy was 85.7% (30/35), which was highly consistent with ultrasound diagnosis (Kappa=0.71, *P* < 0.001) (21);
Combined Diagnostic Value: After combined analysis of synchronous monitoring and echocardiography, the final diagnostic consistency rate was increased to 94.9% (56/59).

**Table 4:** Diagnostic performance of EMAT and LVST for pediatric VSD (echocardiography as gold standard).

Indicator	AUC (95% CI)	Optimal Cut-off Value	Sensitivity (%)	Specificity (%)
EMAT (ms)	0.8560 (0.781∼0.931)	66.30 ms	91.43	91.67
LVST (ms)	0.8774 (0.809∼0.946)	269.38 ms	88.57	70.83
EMAT (ms) – Subtype differentiation (Membranous vs. Muscular)	0.812 (0.654∼0.970)	68.00 ms	84.21	81.25

AUC, area under the receiver operating characteristic curve; CI, confidence interval. Optimal cut-off values determined by Youden index (sensitivity + specificity - 1). Subtype differentiation results are exploratory due to small sample size.

## 4 Discussion

### 4.1 Core value of synchronous PCG-ECG monitoring in VSD differentiation

The clinical manifestations of VSD are diverse. Especially for small muscular defects or infants, they may only present with mild murmurs or even no symptoms, and traditional auscultation is prone to missed diagnosis (20). Through “synchronous analysis of electrical-mechanical activity”, synchronous PCG-ECG monitoring reveals the hemodynamic essence of VSD: during left ventricular systole (QRS complex), high-pressure blood flows through the defect and ejects into the right ventricle, forming characteristic systolic murmurs, and the onset time of the murmur is directly related to the ventricular ejection phase.

In this study, EMAT, LVST, EMAT%, and LVST% in the VSD group were significantly higher than those in the non-VSD group (*P* < 0.001), and the above indicators in the membranous VSD group were significantly higher than those in the muscular VSD group (*P* < 0.05). The AUC values of EMAT and LVST were 0.8560 and 0.8774, respectively, falling within the clinically practical range of 0.85–0.88, which are “good” diagnostic indicators (22). Clinical Meaning of Small Absolute Differences (5–6 ms) in EMAT/LVST: Although the absolute differences in EMAT (≈5–6 ms) and LVST (≈5–7 ms) between groups are small in magnitude, they are clinically meaningful and reproducible for the following reasons: (1) The PCG-ECG device used in this study has a high temporal resolution (500 Hz sampling rate, time resolution of 2 ms), enabling accurate detection of small time-domain differences; (2) All measurements were averaged from 10 consecutive cardiac cycles and normalized to the RR interval, reducing random variability and ensuring the reproducibility of small differences in routine practice; (3) In pediatric cardiology, small changes in electro-mechanical coupling time (e.g., < 10 ms) are associated with subtle structural and hemodynamic abnormalities in congenital heart disease, and these differences have been shown to be a valuable screening marker in previous digital auscultation studies35,37; (4) The combination of EMAT/LVST with murmur characteristics (intensity, timing) further enhances the clinical utility of these small absolute differences, as the electro-mechanical changes are consistent with the pathological murmur features of VSD subtypes.

Children with membranous VSD had higher murmur intensity (mainly grade 4/6∼6/6) and more significant synchrony with the QRS complex (88.9% started 0∼10 ms after QRS), which is consistent with the pathological mechanism of “high-speed blood flow impacting the right ventricular wall due to massive left-to-right shunting" (23); while muscular VSD, due to defects mostly located within the myocardium, has relatively slow shunting speed, and murmurs are mostly soft (grade 1∼2/6) or hidden, but synchronous PCG-ECG indicators can still effectively distinguish them (24).

The anatomical location of membranous VSD (adjacent to the aortic valve, atrioventricular valve annulus, and membranous septum) leads to unique electro-mechanical coupling abnormalities that are not present in muscular VSD, which is the fundamental reason for prolonged EMAT/LVST (independent of defect size/PH):

Abnormal ventricular activation sequence: The membranous septum is the key site for the propagation of the ventricular electrical activation wavefront (bundle branch conduction). Membranous VSD causes local myocardial structural discontinuity, which delays the conduction of the electrical activation signal from the interventricular septum to the left ventricular free wall, resulting in a prolonged interval from QRS onset (electrical activation) to mitral valve closure (mechanical contraction) (i.e., EMAT) (25). Muscular VSD is located in the myocardial septum, far from the main conduction bundle, and does not interfere with the normal ventricular electrical activation sequence.

Valvular and myocardial mechanical load asymmetry: Membranous VSD is adjacent to the aortic and tricuspid valve annuli; even small/medium-sized defects cause asymmetric mechanical tension on the left ventricular outflow tract and atrioventricular valve apparatus during systole, which delays left ventricular systolic termination and mitral/aortic valve closure, leading to prolonged LVST. Muscular VSD is located in the middle/apex of the ventricular septum, and the shunt flow only acts on the right ventricular myocardial wall, without affecting the valvular mechanical load and left ventricular systolic timing.

Mild PH in membranous VSD is a consequence rather than a cause of abnormal electro-mechanical activity: The mild PH in our membranous VSD cohort is secondary to the large shunt volume caused by anatomical location (membranous VSD has a shorter shunt path and higher shunt velocity, even with the same size as muscular VSD), not the direct cause of EMAT/LVST prolongation. Our subgroup analysis has confirmed that PH does not further prolong EMAT/LVST, and VSD subtype is an independent predictor of electro-mechanical indicators.

The PSM analysis further verified that even after balancing defect size and PH status, membranous VSD still showed significant electro-mechanical activation delay, indicating that anatomical location (rather than shunt size or hemodynamic burden) is the core driver of EMAT/LVST prolongation.

Measurement Variability of EMAT% and LVST% (Clarification): The relatively small standard deviations of EMAT% and LVST% in this study (0.01–0.03) are not unusual and are attributed to two key methodological steps: (1) RR interval normalization: EMAT% and LVST% are calculated as the ratio of the time parameter to the total cardiac cycle length, which eliminates the major source of variability—heart rate fluctuations—in the pediatric population (age range 1 month to 16 years); (2) Averaging of 10 consecutive cardiac cycles: We excluded ectopic beats and noisy cycles and used the average value of 10 normal cardiac cycles for each patient, which significantly reduces inter-cycle variability of time-domain parameters. Additionally, the PCG-ECG device has high measurement reproducibility (inter-device r = 0.90–0.92 vs. gold standard systems), further reducing random measurement error. All these steps ensure the reliability of the low variability of EMAT% and LVST% in this cohort.

### 4.2 Complementarity with echocardiography

Although echocardiography remains the unquestionable gold standard for VSD diagnosis and subtype classification—with unparalleled ability to visualize cardiac structure, measure defect size, and assess hemodynamic severity—its application is limited by the need for specialized equipment, trained operators, and patient cooperation (especially in young children) (26). In contrast, PCG-ECG monitoring is designed as a first-line screening/triage tool rather than a diagnostic replacement for echocardiography, with unique advantages that address the limitations of echocardiography in primary care and resource-limited settings: ① Non-invasive, convenient, and low-cost (no need for specialized operators), suitable for large-scale preliminary screening or community-based pediatric heart disease surveys (27); ② Portable and wearable, enabling bedside monitoring and home follow-up without restricting the child's position; ③ Rapid data acquisition and analysis (≤5 min per patient), suitable for triage in pediatric emergency departments to identify children with suspected VSD who require further echocardiographic evaluation; ④ Through qualitative analysis of murmur timing/intensity and quantitative analysis of EMAT/LVST, it can quickly indicate the presence of abnormal ventricular-level shunting, with high sensitivity (91.4%) for VSD detection, ensuring that few true VSD cases are missed in screening.

In this study, the overall sensitivity and specificity of synchronous monitoring for VSD were 91.4% and 87.5%, respectively, both at high levels; the accuracy for differentiating membranous and muscular subtypes was 85.7%, which was slightly lower than that of ultrasound, but the final diagnostic consistency rate was increased to 94.9% through combined analysis, reflecting the complementary value of the two (28). Among them, EMAT had a sensitivity and specificity exceeding 91%, with better balanced performance, suitable for accurate diagnosis; LVST had a sensitivity of 88.57%, suitable for large-scale screening in primary care.

### 4.3 Limitations and future directions

This study still has certain limitations: (1) Small sample size and single-center design: The study included only 59 children (35 VSD), and subgroup analysis for membranous (*n* = 19) and muscular (*n* = 16) VSD was exploratory with relatively small numbers, limiting the statistical power for ROC cut-off value validation and generalizability of the results. Future multicenter prospective studies with larger sample sizes (≥200 VSD patients) are needed to validate the optimal cut-off values of EMAT/LVST and confirm the findings in more diverse pediatric populations (29); (2) Insufficient combination of qualitative and quantitative analysis: although indicators such as EMAT and LVST were quantified, subtype differentiation still partially relies on qualitative murmur judgment (30); (3) Sensitivity to tiny defects needs verification: small muscular defects with diameter <3 mm may have no obvious indicator changes, requiring further verification with expanded samples (31); (4) Infants aged <1 year were not included, and the manifestations of VSD in this population may be different (32). (5) This study initially noted the potential confounding of defect size and mild pulmonary hypertension on EMAT/LVST indicators, and has addressed this issue through stratified and correlation analyses; future large-sample studies can further validate these findings in cohorts with more balanced defect sizes between membranous and muscular VSD subtypes. (6) Lack of long-term follow-up: This study only evaluated the cross-sectional diagnostic value of PCG-ECG, and future studies are needed to investigate the value of EMAT/LVST in monitoring the natural course of VSD (e.g., spontaneous closure of muscular VSD) and evaluating the efficacy of interventional/surgical treatment.

In the future, the sample size can be further expanded, and artificial intelligence technology (such as automatic heart sound signal analysis algorithms) can be combined to improve the objectivity and accuracy of indicator interpretation (33). Additionally, the application value of synchronous monitoring in VSD postoperative follow-up (such as the recovery of EMAT and LVST after shunt closure) can be explored (2). We also plan to validate the PCG-ECG device and its indicators in a multicenter cohort including infants aged <1 year and children with tiny VSD defects, to further improve the clinical applicability of this technology.

## 5 Conclusions

Indicators such as EMAT and LVST from synchronous PCG-ECG monitoring—after normalization to the RR interval to eliminate heart rate interference and adjustment for confounding factors (defect size, hemodynamic burden)— have good diagnostic value for pediatric VSD (AUC 0.8560–0.8774) and can independently differentiate membranous and muscular subtypes (34), with the small absolute differences (5–6 ms) in these indicators being clinically meaningful and reproducible in routine practice due to the high temporal resolution and standardized measurement of the device. The low variability of EMAT% and LVST% is a result of RR interval normalization and averaging of multiple cardiac cycles, ensuring the reliability of these ratio parameters in a wide age range of pediatric populations.

As a non-invasive, convenient, and low-cost screening/triage tool, PCG-ECG monitoring is not intended to replace the gold standard echocardiography but serves as an important supplement to it, with significant clinical application prospects in primary medical care preliminary screening, large-scale population pediatric heart disease physical examinations, and bedside triage in emergency departments. Future multicenter large-sample studies are needed to validate the exploratory ROC cut-off values and further improve the diagnostic accuracy of PCG-ECG for VSD subtypes.

## Data Availability

The raw data supporting the conclusions of this article will be made available by the authors, without undue reservation.
